# Experiences with the implementation of Individual Placement and Support for people with severe mental illness: a qualitative study among stakeholders

**DOI:** 10.1186/s12888-018-1729-4

**Published:** 2018-05-24

**Authors:** Miljana Vukadin, Frederieke G. Schaafsma, Marjan J. Westerman, Harry W. C. Michon, Johannes R. Anema

**Affiliations:** 1 Department of Public and Occupational Health, Amsterdam Public Health research institute, VU University Medical Center, Van der Boechorststraat 7, 1081 BT Amsterdam, the Netherlands; 2 Research Center for Insurance Medicine: collaboration between AMC– UMCG – UWV – VUmc, Amsterdam, the Netherlands; 3 Department of Health Sciences, Vrije Universiteit Amsterdam, De Boelelaan 1105, 1081 HV Amsterdam, the Netherlands; 4 Trimbos Institute, The Netherlands Institute of Mental Health and Addiction, Da Costakade 45, 3521 VS Utrecht, the Netherlands

**Keywords:** Individual placement and support, Implementation, Strategy, Barriers, Facilitators

## Abstract

**Background:**

Individual Placement and Support (IPS) is an evidence-based approach to help people with severe mental illness achieve competitive employment. This article provides insight into an organizational and a financial implementation strategy for IPS in the Netherlands by exploring the perceived facilitators and barriers among participating stakeholders. The goal of this multifaceted strategy was to improve IPS implementation by improving the collaboration between all organizations involved, and realising secured IPS funding with a ‘pay for performance’ element.

**Methods:**

A qualitative, explorative study among practitioners (*n* = 8) and decision makers (*n* = 7) in mental health care and vocational rehabilitation was performed using semi-structured interviews to collect rich information about the possible facilitators and barriers with regard to the organizational and financial implementation strategy for IPS.

**Results:**

Important perceived facilitators were the key principles of the IPS model, regular meetings of stakeholders in mental health care and vocational rehabilitation, stakeholders’ experienced ownership of IPS and collaboration, the mandate and influence of the decision makers involved and secured IPS funding. Important perceived barriers included the experienced rigidity of the IPS model fidelity scale and lack of independent fidelity reviewers, the temporary and fragmented character of the secured funding, lack of communication between decision makers and practitioners and negative attitudes and beliefs among mental health clinicians. Changes in legislation were experienced as a facilitator as well as a barrier.

**Conclusions:**

The results of this study suggest that the collaboration and IPS funding were experienced as improved by applying an organizational and a financial implementation strategy. However, considerable effort is still necessary to overcome the remaining barriers identified and to make the implementation of IPS a success in practice.

**Electronic supplementary material:**

The online version of this article (10.1186/s12888-018-1729-4) contains supplementary material, which is available to authorized users.

## Background

Despite the importance of employment for people with severe mental illness (SMI) [1–4], their labour market participation is poor: in both the United States and Europe it does not exceed 20%. Although between 30 and 65% of these individuals report to desire some form of employment [5–7], they often rely on social assistance or disability benefits [4]. In the Netherlands, for example, up to 25% of the individuals who are granted a disability benefit have SMI [4, 8]. Until now, most vocational approaches for people with SMI have been stepwise, first training individuals before placing them in, often sheltered or volunteer, work (“train and place”) [9, 10]. However, in the past few years, the focus has shifted to supported employment (“place and train”), aiming to place individuals in regular competitive jobs without prevocational training [9–11]. Several systematic reviews conclude that supported employment is more effective than other interventions in obtaining and maintaining employment for people with SMI [12, 13].

Individual Placement and Support (IPS) is an evidence-based example of such an approach [14]. IPS includes the following key principles: eligibility based on client choice (zero-exclusion), a focus on competitive employment and clients’ preferences, work incentives planning, systematic job development, rapid job search and placement with individualized job supports, and integration of mental health and employment services [5]. Fidelity to the IPS model is associated with greater effectiveness with regard to employment outcomes [12, 15]**.**

Despite the strong evidence base for IPS, implementation of this model in the daily practice of mental health care and vocational rehabilitation institutes is difficult [16, 17]. Important barriers to implementation are insufficient collaboration between the organizations involved and inadequate, fragmented and bureaucratically complicated funding [5, 10, 17–20].

To improve IPS implementation, a Dutch mental health agency (MHA), the Dutch Social Security Institute: the Institute for Employee Benefits Schemes (UWV), the municipality of Amsterdam, and a health insurance company (HIC) started to collaborate since 2014. This collaboration included an organizational and a financial strategy to help remove the aforementioned barriers. The organizational implementation strategy consisted of regular meetings between the different stakeholders involved. The financial implementation strategy consisted of secured IPS funding with a ‘pay for performance’ element, rewarding the MHA for placing an IPS participant in a competitive job.

Although previous research has shown that some organizational and financial factors are important implementation barriers [5, 10, 17–20], no studies have provided in-depth understanding of whether strategies focusing on removing these barriers can actually be effective for IPS implementation in practice. The aim of the present study was to provide more insight into an organizational and a financial implementation strategy for IPS, by.

exploring the facilitators and barriers perceived by participating stakeholders.

## Methods

### Study design

A qualitative explorative study among stakeholders was performed using semi-structured interviews to collect rich information about the possible facilitators and barriers with regard to an organizational and a financial implementation strategy for IPS [21].

### Context information

#### IPS before the start of the collaboration

Before this collaboration between the different organizations started, there were few mental health agencies in the Netherlands that provided IPS services according to the IPS model [5]. There were also no formal agreements related to the practical execution and funding of IPS. Depending on the mental health agency, IPS services were usually partly financed by health insurance companies or one of the benefit agencies. In practice, it was rather unclear which part was financed by which organization during an IPS trajectory. This became even more unclear when the client actually started working in a competitive job, and as a consequence lost (a part of) his benefits.

#### IPS within the collaboration

Any client with SMI who received treatment at the MHA and benefits from UWV or the municipality of Amsterdam, could express his desire to obtain a competitive job to his mental health clinician. The client then was referred to an IPS specialist who was part of the same specialized MHA treatment team and provided IPS services according to the IPS model [5]. In the first year of the collaboration each full-time IPS specialist involved had a caseload of 20 clients or fewer and worked within one or two specialized MHA treatment teams. At intake, the IPS specialist and the client decided within eight consultations whether IPS was the right intervention for the client. Then the IPS specialist discussed in a multidisciplinary meeting with vocational rehabilitation practitioners of UWV and the municipality whether the IPS applicant qualified for funding. This meeting and the funding were part of the organizational and financial implementation strategy, respectively.

#### Organizational implementation strategy

The organizational implementation strategy consisted of collaboration between the different organizations involved at two levels:

1. At the management level, there was a meeting every 8 weeks between the decision makers who were considered key leaders and had a managing or advising role within their organization. They initiated the collaboration and arranged the agreements related to the practical execution and funding of IPS. Their goal was to improve the collaboration and communication between the MHA, UWV, the municipality and the HIC, facilitate practitioners, create support within their own organization and ensure IPS sustainment.
2. At the practitioner level, there was a meeting every 6 weeks between the IPS specialists, the labour experts, the insurance physician and the case manager. In their meetings, these mental health care and vocational rehabilitation practitioners discussed whether new IPS applicants qualified for funding. They also discussed the progress of the current IPS participants and any questions related to the participants’ benefits.

#### Financial implementation strategy

The financial implementation strategy consisted of secured IPS funding with a ‘pay for performance’ element. A fair or good IPS fidelity score was a condition for this funding. The duration and amount of the funding (excluding intake and job coaching) depended on the type of benefits the client received.

For clients who received social assistance benefits, IPS was funded by the municipality conform regular responsibilities. The MHA received 900 euro at the start, 900 euro after 3 months and 900 euro after a maximum of 18 months. To stimulate a successful IPS trajectory, the MHA received an extra 1800 euro when placing a client within 9 months in a competitive job for at least 12 h a week during at least 1 month.

For clients who received disability benefits, IPS was funded by UWV conform regular responsibilities. The MHA received 2420 euro at the start and 2420 euro after a maximum of 36 months. To stimulate a successful IPS trajectory, the MHA received an extra 1210 euro when placing a client in a competitive job for at least 12 h at 35% of the minimum wage during at least 2 months.

All the IPS intakes were funded by the HIC for a maximum of 8 hours. All the job coaching was funded by UWV as usual.

#### Socio-political context: The participation act

Since 2015 a new law in the Netherlands, the so called Participation Act, replaced several older Acts for social assistance benefits and disability schemes [22, 23]. This new Act was introduced to stimulate more people with a distance to the labour market, such as people with a disability, into competitive employment. Municipalities were made fully responsible for the execution of the Participation Act. Following the implementation of this Act, both employers and the government guaranteed 125.000 additional jobs by 2026 for people with a disability or social assistance benefits.

### Study participants

All stakeholders involved in the first year of this collaboration were asked to participate in this qualitative study. These stakeholders were from the various organizations: one municipality, two different UWV front offices, two different locations of one MHA and one HIC.

### Interviews

For the interviews (*n* = 15) a topic list was used, based on the theoretical framework of determinants of innovations [24–26]. The interview topics were related to the innovation, the professionals and the organizations involved, and the socio-political context [24–26]. In the present study, the innovation consisted of both IPS and the organizational and the financial implementation strategy. Additional file 1 provides an overview of the interview topics and questions.

The semi-structured interviews were conducted between October 2015 and June 2016 by one researcher (M.V.), trained in qualitative research methods. Participants were asked to tell the interviewer about their experiences with the collaboration with the stakeholders of the other organizations involved, the IPS funding, IPS within the context of this collaboration and the impact of laws and regulations. They were also asked about how the new strategy fitted within their own organization and their role in this collaboration. To elicit any information the participants deemed important, open narrations were encouraged .

Interviews lasted about 1 hour (range 30–95 min) and were voice-recorded. One interview was conducted by telephone; all others were face to face and took place at a location convenient for the participants, usually the participants’ work location.

### Analysis

All interviews were transcribed verbatim. Atlas.ti software was used to facilitate data management and analysis. All transcripts were read thoroughly and analysed. A summary of each interview was made and sent to each participant to determine whether the themes were appropriately described and matched their responses. This member checking was used to improve the credibility and validity of the data [21]. Ten participants responded, and five of them requested minor changes. Thematic content approach was used for data analysis [27].

The analyses were conducted iteratively allowing emerging themes to be explored in subsequent interviews.

All transcripts were coded by one researcher (M.V.). The five most information-rich interviews were coded independently by two researchers (M.V. and F.G.S.). A coding scheme was developed by these two researchers and consensus was reached by discussion. The themes, facilitators and barriers identified by these two researchers were discussed in meetings with a third researcher (M.J.W.), focusing on understanding the collected data and correct interpretation. After several research meetings, a thematic map was developed. Within the themes, facilitators and barriers were distinguished. The aforementioned theoretical framework was used to guide the analysis of the interviews [25]. In the last phase, the themes were refined and the facilitators and barriers identified were sorted and collated according to overarching themes by M.V. and F.G.S. Provisional and final results were critically discussed in the research team meetings with all authors.

The quotations in the Results section were translated from Dutch to English by M.V., and were also discussed with the other authors. Back translation was not performed.

### Ethical considerations

The Medical Ethics Committee of the VU University Medical Center gave approval for the study. All procedures performed in this study were in accordance with the ethical standards of this institutional research committee and with the 1964 Helsinki declaration and its later amendments or comparable ethical standards.

## Results

### Participant characteristics

All invited stakeholders were willing to participate. Participant characteristics are presented in Table 1.

**Table 1:** Participant characteristics

Level (n) Organization Stakeholder	Experience in current role, years
Decision maker (*n* = 7)	
MHA	
Director	7
Policy adviser	8.5
IPS program leader	1
Staff member/ occupational therapist	36
UWV	
Manager	2.5
Municipality of Amsterdam	
Participation adviser	10
HIC	
Mental health care adviser	7
Practitioner (n = 8)	
MHA	
IPS specialist (a)	2
IPS specialist (b)	2
IPS specialist (c)	2
IPS specialist (d)	2
UWV	
Insurance physician	28
Labour expert (a)	15
Labour expert (b)	5
Municipality of Amsterdam	
Case manager	6

*MHA* Mental Health Agency, *UWV* Dutch Social Security Institute: the Institute for Employee Benefits Schemes, *HIC* Health Insurance Company

### Experiences, facilitators and barriers

The participants shared their experiences on how they had perceived this first year of collaboration for IPS between the organizations and mentioned a large number of facilitators and barriers. The perceived facilitators and barriers were classified into different themes related to the innovation and the socio-political context [24–26, 28]. [Additional file 2: Table S2] provides a thematic overview of all perceived facilitators and barriers. A summary of these facilitators and barriers is shown in Table 2. The most discussed themes are reported below, along with quotations to illustrate some facilitators and barriers.

**Table 2:** Summary of perceived facilitators and barriers

Themes	Facilitators	Barriers
1. Innovation		
1.1 IPS		
Intervention	Evidence based effectiveness of IPS	Costs of IPS
	Key principles of the IPS model	IPS model fidelity scale and fidelity reviews
		Compatibility of IPS with existing work procedures
1.2 Collaboration		
Between organizations involved^a^	Regular meetings of stakeholders	Clients’ privacy and medical confidentiality
	Sharing information, knowledge and expertise	Organization of the structural meetings
	Pre-existing relationships and collaboration between stakeholders	Lack of involvement of practitioners in vocational rehabilitation
	Shared interests, goals and vision of stakeholders	Lack of communication between decision makers and practitioners
Professionals involved		
Stakeholders characteristics	Mandate and influence of decision makers	
Attitude and beliefs	Ownership of IPS and collaboration	IPS not experienced as part of the mental health treatment
		Work not experienced as a achievable goal for people with SMI
1.3 IPS funding		
Secured funding^b^	Substantial funding for IPS	Fragmented funding
		Lack of clarity with regard to costs of IPS services
Ethics		
Pay for performance^b^	Pay for performance might encourage IPS specialists	Not appropriate to receive extra payments within health care setting
Sustainability	Covenant between involved organizations stimulates collaboration and funding	Lack of proven cost-effectiveness of IPS Temporary financial agreements between the organizations involved
2. Socio-political context		
Government	Support and funding of Ministry of Social Affairs and Employment New Participation Act provides sense of urgency regarding participation of people with SMI	Dutch social safety net does not stimulate participation in paid work New Participation Act has unwanted consequences Health insurance act limits IPS funding by health insurance company

^a^Part of the organizational implementation strategy. ^b^Part of the financial implementation strategy

#### Innovation

##### IPS

In general, IPS was considered to be an effective intervention for a difficult target group. The key principles of the IPS model were perceived as a facilitator. Several decision makers and MHA practitioners thought implementing IPS with high fidelity to the IPS model was important, because they expected high fidelity IPS services to be more effective.

However, the IPS model fidelity scale was experienced as a barrier by different decision makers, as some items of this scale were considered rigid; the item about integration of the IPS specialist in a specialized MHA treatment team, for example. These decision makers argued that IPS services should not be limited to specialized mental health care, which is now the case in the Netherlands.

MHA decision maker (policy adviser): *“Our healthcare system is continuously changing. That’s a good thing (…) I think you have to keep an open mind for these changes and should not stick to the model fidelity that rigidly.”*

Some MHA decision makers and practitioners also thought it was inappropriate that the fidelity reviews were conducted by the same organization as where IPS specialists were trained. The fidelity reviewers of this organization were not considered independent.

##### Collaboration

The collaboration between the organizations involved was experienced as successful. An important facilitator for this collaboration was having the regular meetings of stakeholders at management and practitioner level. Most participants pointed out that these meetings, particularly at practitioner level, provided designated and easy to reach contact persons. Both collaboration aspects (meetings, contact persons) facilitated short decision lines and fast responses of the stakeholders involved. Some participants also pointed out that regular meetings at both levels stimulated evaluation of new procedures. All participants agreed that regular meetings with stakeholders working within different organizations increased the trust between these stakeholders, as they got to know each other better. This trust improved the perceived reliability of each other’s judgement and facilitated open communication.

MHA practitioner (IPS specialist (a)): *“I considered it a useful meeting (...) the lines of communication are short… and it’s quite useful to have a contact person within those organizations.”*

Most participants pointed out that the collaboration between the organizations was stimulated by stakeholders that experienced ownership of IPS and the collaboration, and were enthusiastic and passionate with regard to IPS. In addition, the mandate and the influence of the participating decision makers was mentioned as an important facilitator by several decision makers. They were seen as opinion leaders with an affinity for the target group, who create support within organizations, arrange funding and promote sustainment of IPS.

MHA decision maker (staff member/ occupational therapist): *“You need people that are inspired (…) with an extreme level of involvement, because otherwise you won’t make it; just procedures aren’t enough. You need people that step up and say: I’m going to do this!”*

Communication between the decision makers and the practitioners was experienced as a barrier by several participants. Most practitioners were not aware of the decisions made during management meetings. Some decision makers also admitted they did not know what the obstacles for MHA practitioners were with regard to IPS and the collaboration with the practitioners of the other organizations.

UWV decision maker (manager): *“No, I don’t know [how these regular meetings between practitioners work in practice] and I suppose that’s strange, because I proposed to initiate these meetings [between practitioners] myself.”*

Some participants, mostly practitioners, also mentioned that there was no formal, written information available about the responsibilities and the roles of the different practitioners involved in the collaboration. This lack of a clear protocol and written information about the agreements between the organizations involved, sometimes resulted in uncertainties and miscommunication among practitioners.

MHA practitioner (IPS specialist (c)): *“I think the agreements between MHA and UWV (…) should be documented, because at the moment there is no written information available.”*

An important barrier mentioned by several MHA decision makers and practitioners was the lack of support experienced within their own organization, based on negative attitudes and beliefs among professionals not directly involved in the collaboration. According to the MHA participants, the mental health clinicians within the specialized MHA treatment teams often did not refer clients to the IPS specialists, because these clinicians did not experience IPS as a part of the regular mental health treatment, and were not focused on recovery related to societal participation in work.

MHA practitioner (IPS specialist (d)): “*Some managers and colleagues see things differently. They don’t support recovery as much as we do within IPS and that is an obstacle (...). [Colleagues argue:] My clients can’t work, my clients won’t work (…). I simply don’t believe that if a case manager has a caseload of 40 clients, none of them wants to work”.*

##### IPS funding

All participants recognized that the secured, substantial funding for IPS was an important facilitator. Several participants thought the pay for performance element of the IPS funding might encourage IPS specialists.

UWV decision maker (manager): *“Maybe some see it as a perverse incentive, but it does provide a reason not to give up for a client if you get a fee for success. Therefore, all in all, I think it’s a very good strategy.”*

Some participants argued that the funding was not adequate, because it was still fragmented and applying for the funding was time consuming. One of the MHA decision makers was also concerned that the funding would not cover all costs of IPS.

MHA decision maker (IPS program leader): *“(…) it’s not just one financial agreement, of course that always creates issues. Ideally, there would be one all-in package [IPS funding] for three years.”*

With regard to the pay for performance element, a MHA decision maker expressed concerns about increased financial risks for the MHA and pressure to place clients in a regular, paid job. A few MHA decision makers also argued it was not appropriate to receive extra payments for achieving goals to place clients in paid work within a health care setting. While some decision makers were afraid that the pay for performance element would be a perverse incentive, other participants were convinced that it had little influence on IPS specialists in daily practice. Most participants thought an important barrier was that the financial agreements between the organizations involved were only temporary.

HIC decision maker (mental health care adviser): “*The municipality has ensured IPS financing for two years, but that means financing ends next year. The same goes for us, we have agreed on financing up to February 2018. In the period ahead, we will all have to discuss how we can ensure sustainable IPS funding.”*

Another barrier mentioned by the decision makers of the benefit agencies and the HIC, was the lack of proven cost-effectiveness of IPS. They all agreed proven cost-effectiveness of IPS was necessary in order to decide whether to continue IPS funding.

#### Socio-political context

The socio-political context was experienced as favourable for the implementation of IPS, but also challenging because of the ongoing changes in laws and regulations regarding the IPS funding and participation of people with SMI. Most participants mentioned the new Participation Act as a facilitator, because it provided a sense of urgency regarding the participation of people with SMI among employers and within benefit agencies. This sense of urgency contributed to the realization of secured IPS funding.

Several participants thought the new Participation Act also had a few unwanted consequences, such as insecurity for people with SMI and organizations involved, and the increased influence of the municipalities regarding the participation policy (decentralization).

MHA practitioner (IPS specialist (d)): *“I think the Participation Act is really complicated, because your access to [IPS] services depends on the municipality you live in. (…) you depend on the political orientation of your municipality, how rich your municipality is and what they want to spend money on. Well, I think that’s insane.”*

## Discussion

The aim of the present study was to gain in-depth insight into an organizational and a financial implementation strategy for IPS by exploring the perceived facilitators and barriers among participating practitioners and decision makers in mental health care and vocational rehabilitation. Using a theoretical framework [24, 25], several perceived facilitators and barriers related to IPS, the implementation strategies and the socio-political context were identified. Important perceived facilitators were the key principles of the IPS model, regular meetings of stakeholders in mental health care and vocational rehabilitation, stakeholders’ experienced ownership of IPS and collaboration, the mandate and influence of the decision makers involved and secured IPS funding. Important perceived barriers included the experienced rigidity of the IPS model fidelity scale and lack of independent fidelity reviewers, the temporary and fragmented character of the secured funding, lack of communication between decision makers and practitioners and negative attitudes and beliefs among mental health clinicians. Changes in legislation were experienced as a facilitator as well as a barrier.

### Comparison with other studies

In the literature on multifaceted implementation strategies, financial and organizational implementation activities are underrepresented [29]. Grimshaw et al. showed that most strategies focused on professionals involved [30]. An important reason for that may be that professional-directed implementation strategies are easier to realise in the study practice than financial or organizational strategies [29]. There is, however, some literature on facilitators and barriers to the implementation and sustainment of supported employment [10, 20, 31], and to components of multifaceted implementation strategies for supported employment [32, 33]. For example, two studies evaluating implementation [10] and sustainment [20] of supported employment found that important facilitators to IPS implementation and sustainment were strong personal commitments by program leaders [10] and leadership [20], in line with the facilitators found in the present study. Unlike the present study, these studies [10, 20] focused only on the experiences of MHA and IPS stakeholders and did not include stakeholders from different organizations.

Regular meetings of professionals comparable to the meetings in this implementation study were also found to be important in the study by Holwerda et al. [34]. Using questionnaires to assess the collaboration between professionals in mental health care and vocational rehabilitation to support employment of individuals with mental disorders, they also found that collaborating in a structural way was essential for developing an effective collaboration between the organizations involved [34].

Although the secured IPS funding (including pay for performance) as a strategy was experienced as a facilitator, it was not perceived as adequate, as the funding itself was still rather fragmented, and the agreements about the funding were only temporary. Previous studies also identified inadequate funding as an important barrier to IPS implementation and sustainment [10, 19, 20, 31]. Noel et al. concluded that, within the context of an active learning community, secured funding was an important facilitator to IPS sustainment [20]. This learning community promotes dissemination, implementation, sustainment and expansion of IPS [19, 20].

The finding of limited consensus about the added value of pay for performance in the present study was also reported by McGrew et al. [32], who found that although some participating professionals where satisfied with the funding, others raised concerns about increased financial risks, pressure to achieve job placements and possible pressures for adverse client selection.

### Strengths and limitations

A strength of this study is that it is one of the first studies to assess the experiences with a multifaceted implementation strategy for IPS among stakeholders. Another strength is that all decision makers and practitioners involved in the first year of the collaboration between the different organizations were interviewed. This helped to achieve an accurate and complete understanding of perceived facilitators and barriers among these different stakeholders. Furthermore, the participants provided feedback on their interview summary, which improves the credibility and validity of the data. The credibility of the analysis is also increased by coding five interviews independently and developing the coding scheme by two researchers, and discussing the results in research team meetings with all authors.

The use of a theoretical framework [24, 25] to develop a topic list and guide the interviews and their analysis, is both a strength and a limitation of this study. It is a strength because using a framework based on prior research enables a structured analysis and might improve the validity of the data; it is also a limitation because the framework [24, 25] focuses on innovations within health care organizations. The innovation in this study, however, consisted of a multifaceted implementation strategy, mainly focusing on improving the IPS implementation by collaboration between different types of organizations and secured IPS funding.

A limitation of this study is the limited generalizability of the findings due to the small number of participants within this qualitative study focusing on the Dutch social security context. However, similar facilitators and barriers to the implementation of IPS have been reported in other countries with a different social security system [19, 20, 31, 35].

### Implications for practice and research

Important barriers were the ignorance of decision makers regarding obstacles for MHA practitioners, and a lack of formal written information about the responsibilities and the roles of the different practitioners involved. These findings suggest that communication between decision makers and practitioners, and information transfer with regard to the innovation, can be improved and therefore need more attention in future implementation strategies in order to make IPS a success in practice.

The perceived barriers related to the IPS funding suggest that there is a need for one, sustainable funding for all clients based on proven cost-effectiveness of IPS. Consequently, future research should focus on evaluating the cost-effectiveness of IPS. In addition, the.

experienced rigidity of the IPS model fidelity scale and lack of independent fidelity reviewers were perceived as barriers to providing IPS services and may need further evaluation in the European context, considering the dependence of IPS funding on the IPS fidelity score. However, it appears to be important to continue IPS fidelity monitoring, since ongoing fidelity monitoring may promote long-term sustainability of IPS [15, 19, 31].

An important barrier was the lack of support experienced within the MHA, based on negative attitudes and beliefs among mental health clinicians. Fortunately, these negative attitudes and beliefs of clinicians are likely to change over time, as they come to better understand the relevance of employment on health for everyone [36]. This process may be accelerated by increasing clinicians’ involvement in the IPS trajectories and by presenting frequently examples of successful IPS candidates to them. However, not only were negative attitudes and beliefs among clinicians experienced as challenging for IPS implementation in the MHA by several MHA participants, the ongoing changes in laws and regulations regarding IPS funding and participation of people with SMI also seemed to complicate this process. It is therefore important to ensure ongoing support and continuity within all organizations involved, and to continue facilitating IPS specialists.

## Conclusions

This qualitative study provides more insight into the perceived facilitators and barriers of an organizational and a financial implementation strategy for IPS in the Netherlands by exploring the experiences of stakeholders involved. Important perceived facilitators were the key principles of the IPS model, regular meetings of stakeholders in mental health care and vocational rehabilitation, stakeholders’ experienced ownership of IPS and collaboration, the mandate and influence of the decision makers involved and secured IPS funding. Important perceived barriers included the experienced rigidity of the IPS model fidelity scale and lack of independent fidelity reviewers, the temporary and fragmented character of the secured funding, lack of communication between decision makers and practitioners and negative attitudes and beliefs among mental health clinicians. Changes in legislation were experienced as a facilitator as well as a barrier. These results suggest that the collaboration and IPS funding were experienced as improved by applying this implementation strategy. However, considerable effort is still necessary to overcome the remaining barriers identified and to make the implementation of IPS a success in practice.

## Additional files

Additional file 1: Experiences with the implementation of Individual Placement and Support for people with severe mental illness: a qualitative study among stakeholders. Overview of interview topics and questions. (DOCX 28 kb)

Additional file 2: Experiences with the implementation of Individual Placement and Support for people with severe mental illness: a qualitative study among stakeholders. **Table S2.** Thematic overview of perceived facilitators and barriers. (XLSX 27 kb)

## Abbreviations

HIC: Health insurance company
IPS: Individual Placement and Support
MHA: Mental health agency
SMI: Severe mental illness
UWV: The Dutch Social Security Institute: the Institute for Employee Benefits Schemes
