# Supplementary material for: Experiences with the implementation of Individual Placement and Support for people with severe mental illness: a qualitative study among stakeholders
Source: BMC Psychiatry. 2018 May 24;18:145. doi: 10.1186/s12888-018-1729-4 (PMC5968490; doi:10.1186/s12888-018-1729-4)
Supplement: Supplementary file 1 — Experiences with the implementation of Individual Placement and Support for people with severe mental illness: a qualitative study among stakeholders. Overview of interview topics and questions. (DOCX 28 kb) [file 12888_2018_1729_MOESM1_ESM.docx]

**Additional file 1: Experiences with the implementation of Individual Placement and Support for people with severe mental illness: a qualitative study among stakeholders.**

**Overview of interview topics and questions^1,2^**

General information about the participant

1. What is your current age?
2. What is your current job function/ role?
3. Since when have you worked in this job function/ role?
4. What is your role in the IPS collaboration?
5. Have you been involved in the implementation of an innovation before?

Collaboration between the MHA, UWV, the municipality and the HIC

1. How have you experienced the collaboration between the MHA, UWV, the municipality and the HIC?
2. In your opinion, have the newly made agreements related to this collaboration made a difference?
3. How satisfied are you with this collaboration in comparison to the situation before this collaboration?
4. Do you have any suggestions to improve future collaboration?

Regular meetings at the management level

1. How did you experience the meetings at the management level? How did these meetings turn out in your opinion?
2. How satisfied are you with these meetings?
3. According to you, what are points for further improvement of these meetings?

Regular meetings at the practitioner level

1. How did you experience the meetings at the practitioner level?
2. How satisfied are you with these meetings?
3. According to you, what are points for further improvement of these meetings?

IPS funding with pay for performance (i.e. financial agreements between 1) the MHA and UWV and 2) the MHA and the municipality)

1. According to you, what are the most important differences and similarities between the two financial agreements made between the participating organisations? For example: the duration of the funding, the total amount of money, the level of pay for performance and the amount of administrative burden (i.e. number and size of required reports)?
2. What do you think of these financial agreements? Have these agreements turned out as planned in practice?
3. What is your opinion on the pay for performance element of the IPS funding?
4. How satisfied are you with the IPS funding?
5. According to you, what are points of improvement regarding the IPS funding? Do you have any suggestions for the future regarding this funding?

Role of the IPS training organization

1. What are your experiences with the IPS training organization?
2. What do you think about the role of this organization?
3. How satisfied are you with the IPS training?
4. How satisfied are you with the fidelity reviews?

Characteristics of the innovation (i.e. IPS within the context of the collaboration between the MHA, UWV, the municipality and the HIC, including financial agreements related to IPS funding)

1. Were the new agreements and procedures related to the IPS collaboration and funding clear to you?
2. Does the innovation fit within the existing work procedures?
3. Do you benefit from the innovation?

Characteristics of professionals (in)directly involved

1. Have you experienced support of your direct colleagues (same job function/ role) with regard to the innovation?
2. Have you experienced support of other professionals (other job function/ role) with regard to the innovation?
3. Have you experienced support of your supervisor/ manager with regard to the innovation?

Characteristics of the organizations involved

1. According to you, to what extent have the following factors influenced the innovation in your own organization?
   - Staff turnover
   - Staff capacity
   - Number of people involved
   - Time available for the innovation

Socio-political context

1. According to you, what is the impact of (changes in) laws and regulations (e.g. the Participation Act) on the innovation?
2. According to you, how do clients feel about innovation?

IPS: Individual Placement and Support

MHA: mental health agency

UWV: the Dutch Social Security Institute: the Institute for Employee Benefits Schemes

HIC: health insurance company

^1^ Fleuren M, Wiefferink K, Paulussen T: Checklist determinanten van innovaties in gezondheidszorgorganisaties. TSG 2010, 88:51-54.

^2^ Fleuren MA, Paulussen TG, Van Dommelen P, Van Buuren S: Towards a measurement instrument for determinants of innovations. Int J Qual Health Care 2014, 26:501-510.
